# Electron traps and energy storage: modeling a bright path to the future

**DOI:** 10.1107/S205252062301003X

**Published:** 2023-11-23

**Authors:** Renaldo T. Moura Jr

**Affiliations:** a Department of Chemistry and Physics, Center of Agrarian Sciences, Federal University of Paraiba, Areia, PB 58397-000, Brazil; b Computational and Theoretical Chemistry Group (CATCO), Department of Chemistry, Southern Methodist University, Dallas, TX 75275, USA

**Keywords:** luminescence, electron trap, doped materials, TDDFT, solid state, lutetium oxide

## Abstract

By employing time-dependent density functional theory for solid-state chemistry, the research presented by Andrii Shyichuk [*Acta Cryst.* (2023), B**67**, 437–449] significantly contributes to the understanding of electron/hole traps in doped materials.

The luminescence phenomenon manifests when a substance emits light without undergoing heating. This occurrence is typically initiated through the excitation of the substance by various forms of energy. Light, serving as an excitation source, is capable of inducing luminescence, and in such cases, the exciters are referred to as phonons, giving rise to what is known as photoluminescence. It is noteworthy that alternative excitation sources exist, as discussed by Kulesza *et al.* (2016 ▸), such as electric current (resulting in electroluminescence), electron beam exposure (leading to cathodoluminescence), energy released during a chemical reaction (resulting in chemiluminescence), mechanical action (causing mechanoluminescence), ionizing radiation (yielding radioluminescence), and even changes in temperature, either through heating (thermoluminescence) or cooling (cryoluminescence), among others.

In all instances, the excitation source serves the purpose of promoting electrons that, within a brief period, undergo recombination, thereby giving rise to the luminescence phenomenon. Notably, materials exhibiting an extended duration of glow subsequent to the cessation of excitation are characterized as having persistent luminescence (Xu & Tanabe, 2019 ▸). The gradual release of excited electrons can be classified as a form of energy storage. This particular phenomenon involves the presence of energy traps, such as electron or hole traps, within a material, which are occupied during the excitation process. Understanding this phenomenon poses a challenge due to the conventional treatment of solid-state systems under the non-localized regime of conduction and valence band structures. Consequently, conceiving of a trap that completely localizes electrons becomes difficult in this context.

The challenge of localization is not novel in solid-state chemistry. In 1997, Marzari and colleagues introduced the Maximally Localized Wannier Functions (MLWF) method, presenting an alternative avenue for analyzing chemical bonding in periodic systems (Marzari & Vanderbilt, 1997 ▸; Silvestrelli *et al.*, 1998 ▸; Souza *et al.*, 2001 ▸; Marzari *et al.*, 2012 ▸). MLWFs replace the traditional non-localized Bloch wavefunctions, serving to localize the band structure into MLWF orbitals. Additionally, the Crystal Orbital Hamiltonian Population (COHP) method (Dronskowski & Bloechl, 1993 ▸) and the Crystal Orbital Overlap Population (COOP) method (Hughbanks & Hoffmann, 1983 ▸) offer an alternative means of characterizing chemical bonds in periodic systems (Ruggiero *et al.*, 2015 ▸). These two techniques share almost identical formulations and operate in a similar way to conventional Density of States (DOS) calculations. However, instead of considering contributions from all orbitals in a molecular or periodic system, they facilitate the examination of specific interaction pairs between two sets of orbitals (Ruggiero *et al.*, 2017 ▸). Both COOP and COHP schemes allow for the quantification of orbital overlap and the strength of interatomic bonds (Rohling *et al.*, 2019 ▸). These methods provide valuable insights into the bonding characteristics of periodic systems, delivering a detailed analysis of specific orbital interactions and exemplifying effective localization tools for such systems.

The work of Andrii Shyichuk (2023 ▸) (in this issue of *Acta Crystallographica Section B*) is truly inspiring, particularly in its exploration of the localization of electron traps in doped materials. Employing a time-dependent extension of density functional theory – a widely used framework in solid-state chemistry and physics – Shyichuk significantly advances our understanding of the behavior of an oxygen vacancy in cubic lutetium oxide (c-Lu_2_O_3_). The study delves into the impact of dopants such as Hf or Zr on the electron density trapped within the vacancy.

The results presented by Shyichuk offer intriguing and insightful findings, exemplified by the localized behavior of the trapped electron (see Fig. 1 ▸). The study demonstrates that the trapped electron density not only occupies the vacancy site with a semi-local character (as illustrated on the left-hand side of Fig. 1 ▸) but also engages *d*-type orbitals in elements such as Hf (as depicted on the right-hand side of Fig. 1 ▸). This nuanced understanding is thoroughly discussed, shedding light on the multifaceted nature of electron trapping in doped materials.

In the context of phosphors, Yen *et al.* (2007 ▸) emphasize the importance of addressing various facets such as lattice relaxation, charge-compensating defects, intrinsic defects, the nature of the bottom of the conduction band, and the dynamic properties involved in charge localization and delocalization processes, along with theoretical modeling. Moreover, Yen is acknowledged (Kulesza *et al.*, 2016 ▸) for asserting that the conceivable applications of these materials are limited solely by the innovative capacity of individuals engaged in research and development.

In this context, the work of Shyichuk (2023 ▸) is deemed a milestone. This designation is warranted as Shyichuk’s research significantly contributes to the understanding of electron/hole traps in doped materials. By exploring the intricacies of electron/hole traps, Shyichuk’s work extends the boundaries of knowledge, fostering an expanded imagination among readers. This contribution not only advances our comprehension of these materials but also illuminates a promising path toward the development of more energy-efficient materials.

## Figures and Tables

**Figure 1:**
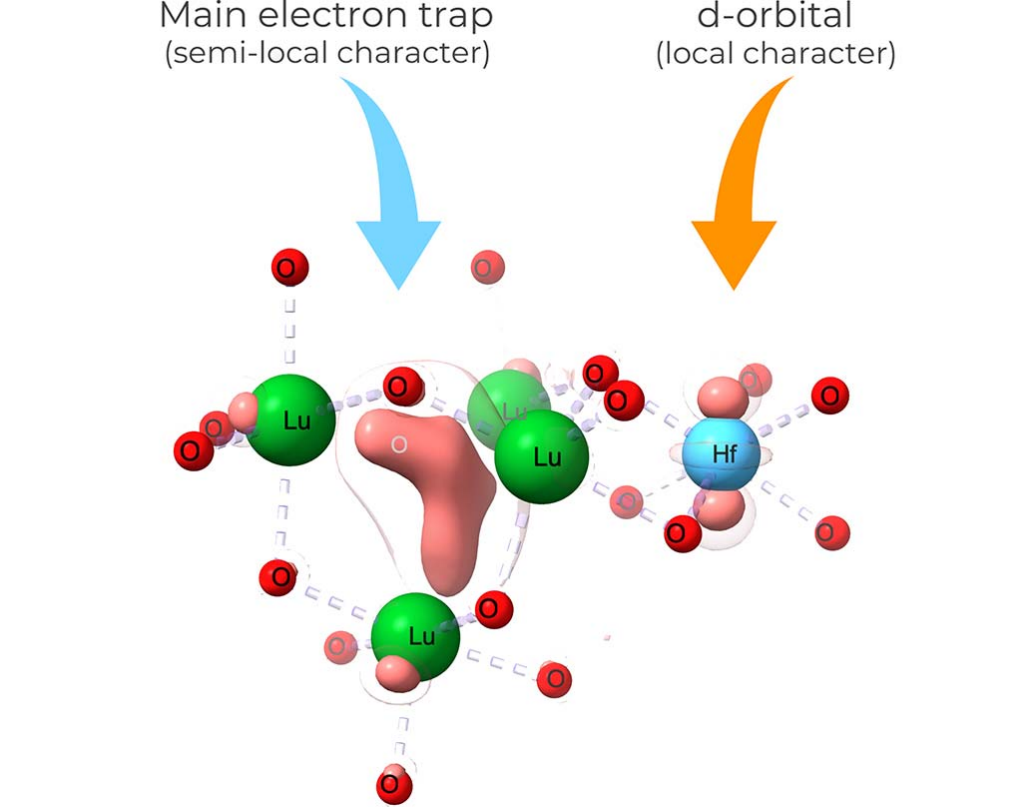
Trapped electron density as obtained by Shyichuk (2023 ▸) for an oxygen vacancy in Lu_2_O_3_:Hf. Isosurfaces have contour values of 0.004 and 0.009 e a^3^_0_. Structure and electron density maps were created using the *ChimeraX* software (Pettersen *et al.*, 2021 ▸; Goddard *et al.*, 2018 ▸).
